# Converting a breast cancer microarray signature into a high-throughput diagnostic test

**DOI:** 10.1186/1471-2164-7-278

**Published:** 2006-10-30

**Authors:** Annuska M Glas, Arno Floore, Leonie JMJ Delahaye, Anke T Witteveen, Rob CF Pover, Niels Bakx, Jaana ST Lahti-Domenici, Tako J Bruinsma, Marc O Warmoes, René Bernards, Lodewyk FA Wessels, Laura J Van 't Veer

**Affiliations:** 1 Agendia BV, Slotervaart Medical Center 9D, Louwesweg 6, 1066 EC Amsterdam, The Netherlands; 2 Netherlands Cancer Institute, department of Molecular Biology, Plesmanlaan 121, 1066 CX Amsterdam, The Netherlands

## Abstract

**Background:**

A 70-gene tumor expression profile was established as a powerful predictor of disease outcome in young breast cancer patients. This profile, however, was generated on microarrays containing 25,000 60-mer oligonucleotides that are not designed for processing of many samples on a routine basis.

**Results:**

To facilitate its use in a diagnostic setting, the 70-gene prognosis profile was translated into a customized microarray (MammaPrint) containing a reduced set of 1,900 probes suitable for high throughput processing. RNA of 162 patient samples from two previous studies was subjected to hybridization to this custom array to validate the prognostic value. Classification results obtained from the original analysis were then compared to those generated using the algorithms based on the custom microarray and showed an extremely high correlation of prognosis prediction between the original data and those generated using the custom mini-array (p < 0.0001).

**Conclusion:**

In this report we demonstrate for the first time that microarray technology can be used as a reliable diagnostic tool. The data clearly demonstrate the reproducibility and robustness of the small custom-made microarray. The array is therefore an excellent tool to predict outcome of disease in breast cancer patients.

## Background

Microarray analysis is a widely used technology for studying gene expression on a global scale. However, the technology is presently not used as a routine diagnostic tool. Various studies have shown that microarray analysis results in improved diagnosis and risk stratification in many cancers [1-12]. More specifically, in human breast cancer molecular profiles have identified subtypes [3,8], and prognostic subgroups that are relevant to patient management [4,6,13,14], and may add to the prediction of therapy response [15-18].

One study involved the discovery of a profile associated with the risk of early development of distant metastasis in young patients with lymph-node negative breast cancer [6]. The development of distant metastases is the primary cause of death in breast cancer patients; approximately one third of women with lymph node negative breast cancer will develop distant metastasis. The challenge therefore is to predict the risk of metastasis at the time of primary diagnosis and accurately manage those patients identified as high-risk. The Amsterdam 70-gene prognosis profile has been shown to outperform all clinical parameters in predicting distant metastasis [13]. The ability to use this profile in a high throughput diagnostic setting would be a great advantage in the prognosis and treatment of breast cancer.

This profile, however, was generated on oligonucleotide microarrays containing approximately 25,000 60-mer oligonucleotides. Using these arrays for clinical practice would not only be costly, but their one-sample-per-chip design would not allow for high throughput processing of many samples on a routine basis. Recently, an 8-pack format with 8 identical sub-arrays, containing a limited number (1900) of 60-mer oligonucleotides became available. This would allow less sample RNA input for labeling and hybridization and data processing time could be substantially reduced, permitting test results to become available within 5 days.

Nonetheless, there are several issues to consider when 'reading' expression profiles from mini-microarrays. Data processing steps, such as normalization to remove systemic variation and background subtraction, may require re-optimization for the smaller number of probes present. Apart from such issues of data processing, the original biological samples used to generate the original profile need to be available for confirmation and validation purposes.

In this paper we describe the development of a customized diagnostic breast cancer mini-array, MammaPrint, based on the Amsterdam 70-gene expression profile [6], and describe its reliable use in a diagnostic setting.

## Results

**Figure 1:**
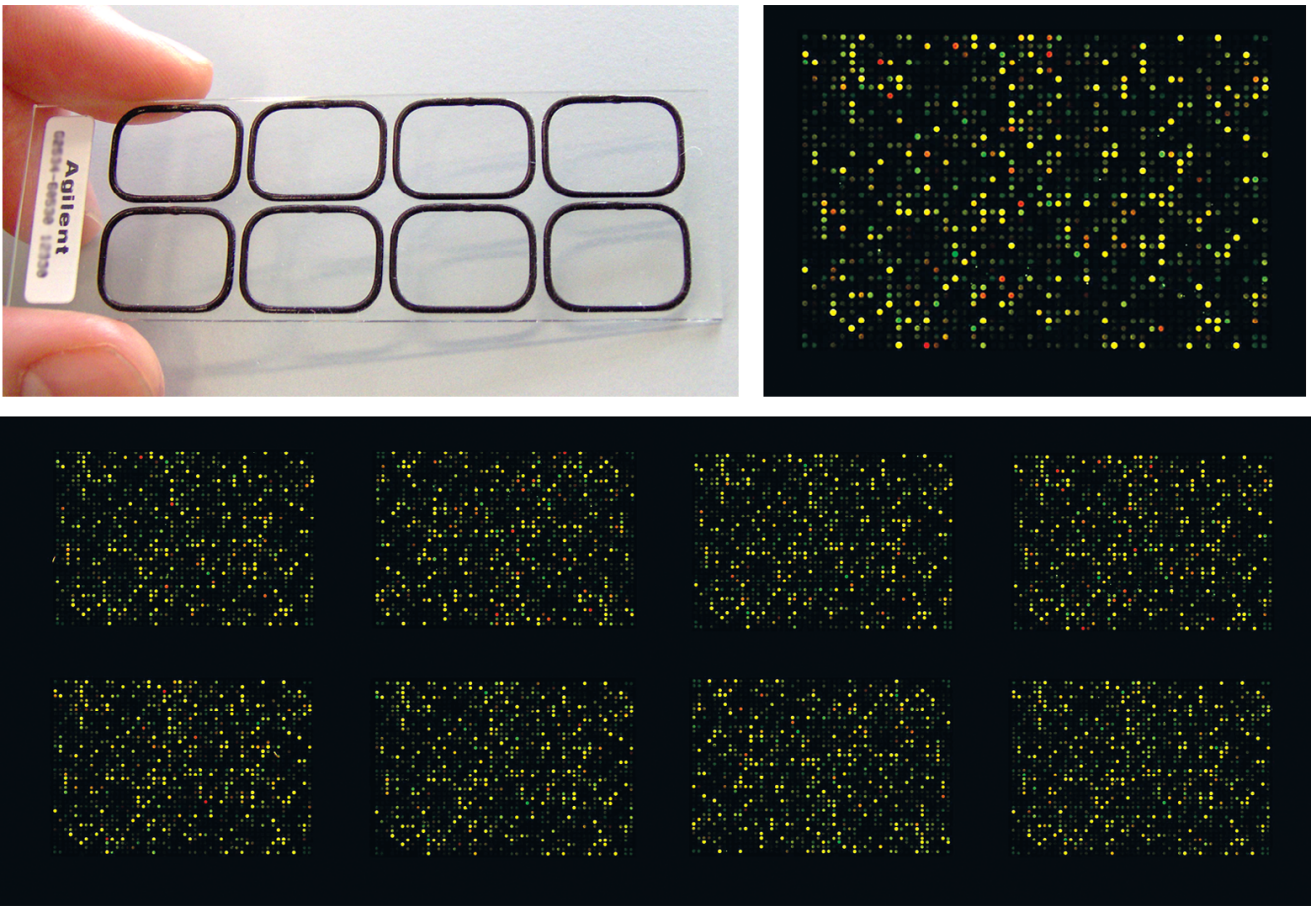
MammaPrint 8-pack, a single 1" × 3" slide containing 8 mini-arrays with 1,900 60-mer oligonucleotide probes, allowing for eight individual hybridizations simultaneously. The samples are hybridized against a common breast cancer reference pool.

Recently, using complex microarrays, a 70-gene prognosis profile was identified that is a powerful predictor for the outcome of disease in young breast cancer patients. This profile was generated using 78 tumor samples of patients having lymph node negative disease by hybridization of fluorescent-dye labeled RNA to microarrays containing 25,000 60-mer oligonucleotide probes. To enable the use of this prognostic classifier in a diagnostic setting, custom-made 8-pack mini-arrays were developed (Agilent Technologies). This mini-array is a single 1" × 3" slide containing eight identically printed regions or sub-arrays, each containing 1,900 60-mer oligonucleotide probes, including the 70 prognostic classifier genes [6]. This allows eight individual hybridizations to be carried out simultaneously on a single microarray slide (Figure 1).

To increase measurement precision, each of the signature genes was spotted in triplicate and an error-weighted average of the intensity ratios was calculated. In the original studies another method was used to decrease uncertainties of the array measurements, i.e., the use of the quantity Xdev [19,20], however, this showed undesirable artifacts since the variance in error estimation is dependent on the number of spots used in the calculations.

To determine if the customized mini microarray test performs as well as the original 25 k microarrays [6,13], RNA of samples used in the original series to develop the 70-gene prognosis classifier [6] were retrieved, labeled and re-hybridized against a common reference sample with reverse fluorescent dyes using the 8-pack mini-arrays. Since different measurement quantities were used (Xdev versus LogRatio), we reconstructed the 'good prognosis template' by using the data of the 44 good outcome patients generated on the mini-array based on log ratios. Disease outcome classification of individual samples was then determined by the cosine correlation to this recreated template in a leave-one-out cross validation procedure.

**Figure 2:**
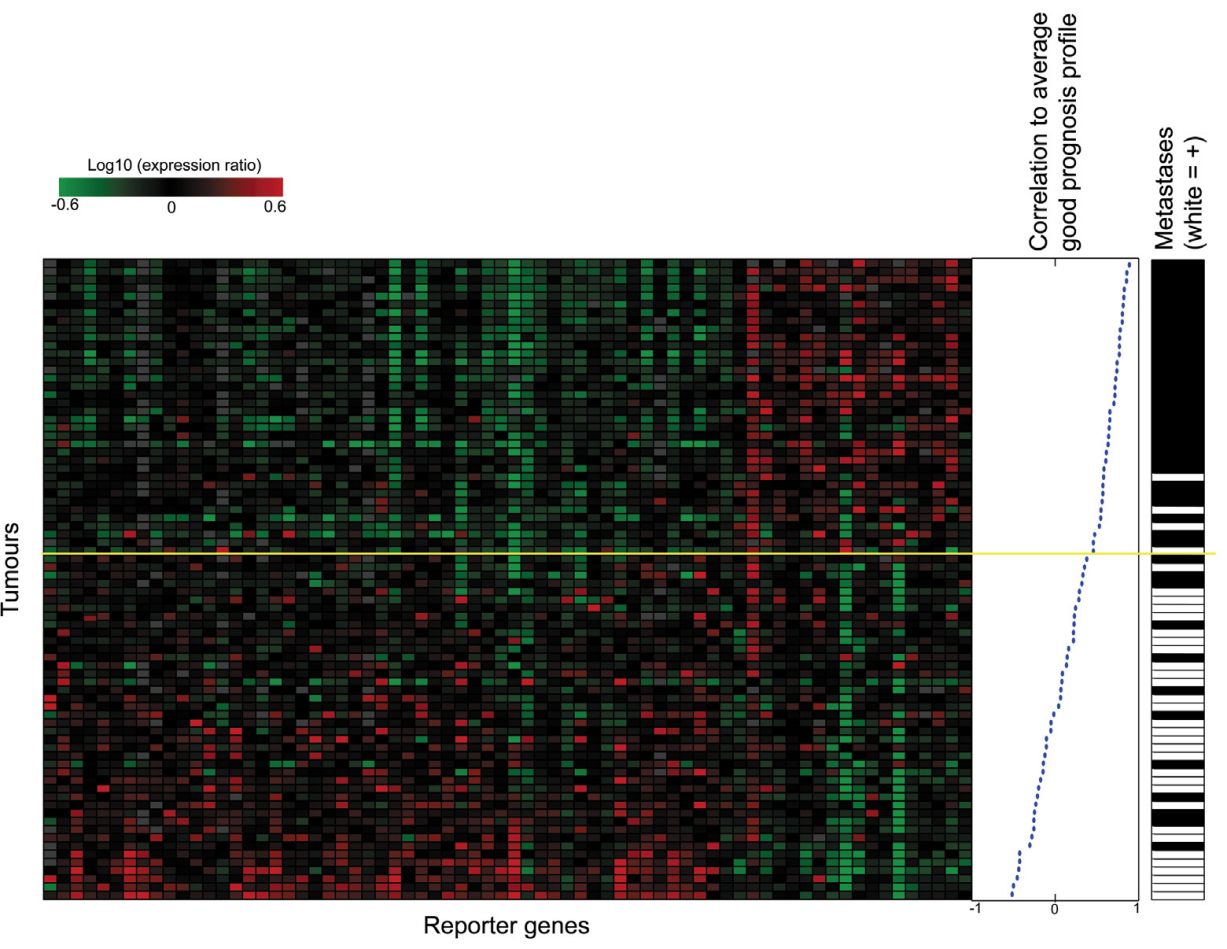
Expression data matrix of 70 prognostic markers genes from tumors of 78 breast cancer patients hybridized using the custom microarray. Each row represents a tumor and each column a gene. Genes are ordered according to their original ordering. Tumors are ordered by their correlation to the average profile of the good prognosis group (middle panel). The metastases status for each patient is shown in the right panel. White indicates patients who developed metastases within 5 years after the initial diagnosis, black indicates patients who continued to be metastasis free for at least 5 years.

The expression intensities of the 70 signature genes for the 78 original samples hybridized to the customized array are shown in Figure 2. The tumors are rank-ordered according to their correlation coefficients with the re-established 'good prognosis template' (Figure 2 middle panel). Genes are ordered according to their correlation coefficient with the two prognostic groups as previously described [6]. Tumors with correlation values above or below the previously determined threshold [6] (indicated by the yellow line in Figure 2) were assigned to the good or poor prognosis profile group, respectively. The right panel in Figure 2 shows the distant metastasis status of the patients and confirms the strong correlation of prediction and high accuracy between the profile predicted and actual outcome of disease of the patients, as observed in the original studies [6,13].

### Comparison to original data

**Figure 3:**
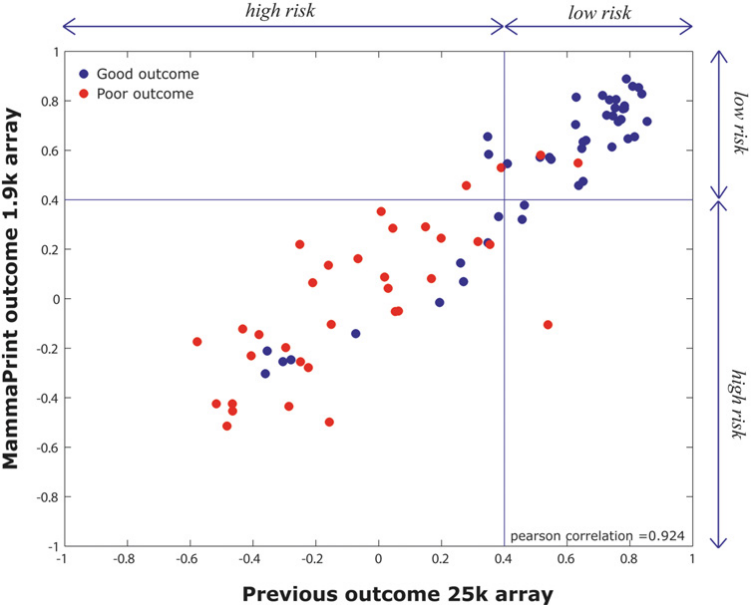
Comparison of current data to published values [6]. Correlation of the 70 genes from each tumor to the average expression profile of the good outcome patients is plotted. On the Y axis results from the customized 8-pack test is plotted and on the X axis results are plotted using published data from the original paper [6] using Xdev values (see text)

To perform a comprehensive evaluation of the mini-array results, we compared in Figure 3 the current classification to the good and poor prognostic profiles with that of the originally published classification for each sample. Results from the original study are shown (X-axis) plotted against those obtained from the customized mini-array (Y axis) [6]. The data generated using the diagnostic test is highly similar (Pearson correlation of 0.92, p < 0.0001) to the original published data. The overall accuracy of the diagnostic test was determined by calculating the odds ratio for the development of distant metastases within five years. The odds ratio calculated based on the current results (OR = 13. 95%CI 3.9 to 44) was highly comparable to the original data (OR = 15, 95%CI 2.1 to 19) using the methods described in the supplementary information of [6].

A more detailed evaluation revealed seven discordant cases between MammaPrint risk assessment and the published data. These cases included two patients that did not develop distant metastases, who were classified as being poor prognosis in the published data [6], but the diagnostic test correctly classified them into the good prognosis group. Furthermore, one patient who did develop metastases was originally classified as good prognosis, whereas in the current results this patient was classified correctly as having a poor prognosis. On the other hand, however, there were two good outcome patients classified as poor prognosis using the diagnostic test, while in the original data these samples were classified correctly, as well as two poor outcome patients classified as good prognosis by the current test who where correctly classified by original analysis as poor prognosis.

### Customized mini-array reproducibility

**Figure 4:**
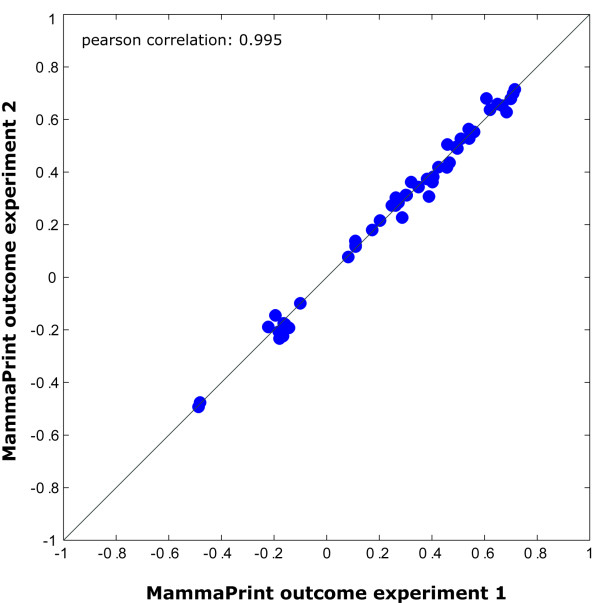
Custom array outcome of replicate experiments. Cosine correlation to the good prognosis template is plotted, and is highly similar between duplicate experiments.

To further investigate if the differences seen were due to technical variation of the current test or could be otherwise explained, 49 samples were amplified and hybridized a second time to the 8-pack mini-array (Figure 4). The Pearson correlation for the replicate experiments was 0.995, indicating a very high degree of reproducibility of classification for individual tumor samples using the customized 8-pack array. Also an ANOVA analysis performed on the 70-gene expression values obtained in the duplicate experiments showed no significant differences, independent of variation between individual samples and profile genes (p = 0.960).

**Figure 5:**
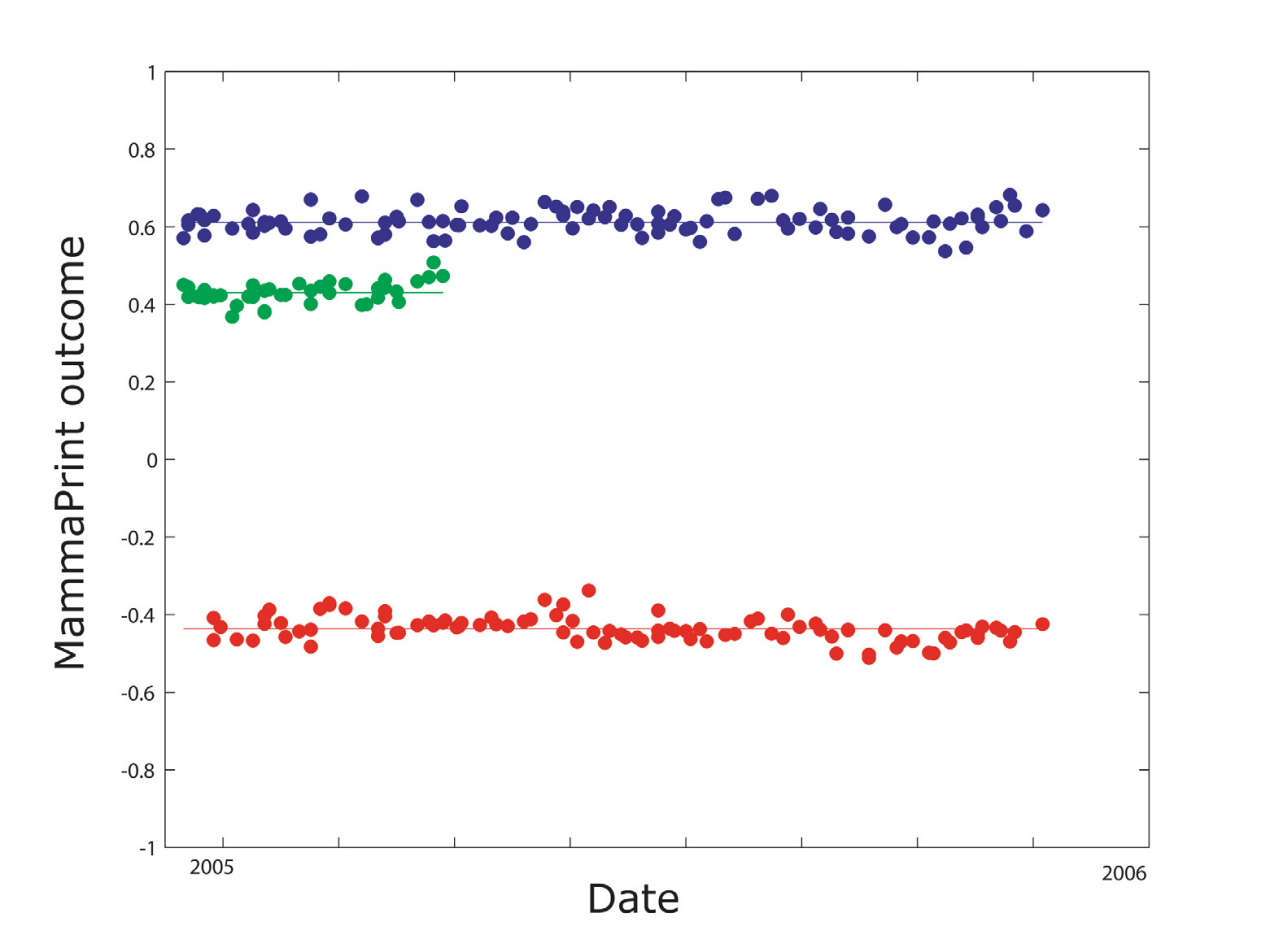
Custom diagnostic microarray outcome of two samples over time. The correlation to the good prognosis profile of three samples (HRC, LRC, and BLS) of >100 measurements over a period of 12 months shows constant outcome.

To ensure that the outcome of the test does not change over time, two samples were amplified and labeled repeatedly over a period of 12 months (Figure 5). One sample (HRC) was classified as poor prognosis with an average cosine correlation to the good prognosis template of -0.44. The other sample (LRC) was classified as good prognosis (average correlation to the good prognosis template of 0.61). Both samples were stable over time as shown and the diagnostic test result was observed to have a very low standard deviation (LRC and HRC stdev 0.028, i.e. technical variation), indicating the robustness of the diagnostic test.

A sample close to the classification threshold was analyzed 40 times in a period of 4 months. The average correlation to the good prognosis template was 0.430 with a standard deviation of 0.027. The sample was misclassified 6 times (15 %), which is in agreement with the expected chance of misclassification (14%) based on the area of the Gaussian that falls on the other side of the decision boundary (s = 0.028 (the overall standard deviation for the test) and m = 0.430).

### Clinical validation study comparison

To more accurately estimate the risk of metastases associated with the 70-gene prognostic profile, a validation study [13] was performed using a cohort of 295 young breast cancer patients. For the current study we selected the 151 patients from this cohort without lymph node involvement at diagnosis of which 145 RNAs were available.

We calculated the probability of a patient remaining free of distant metastases and overall survival according to the prognosis profile and compared this to the published data [13].

Once more the data generated using the customized array is highly similar (Pearson correlation of 0.88, p < 0.0001) to the original data.

**Figure 6:**
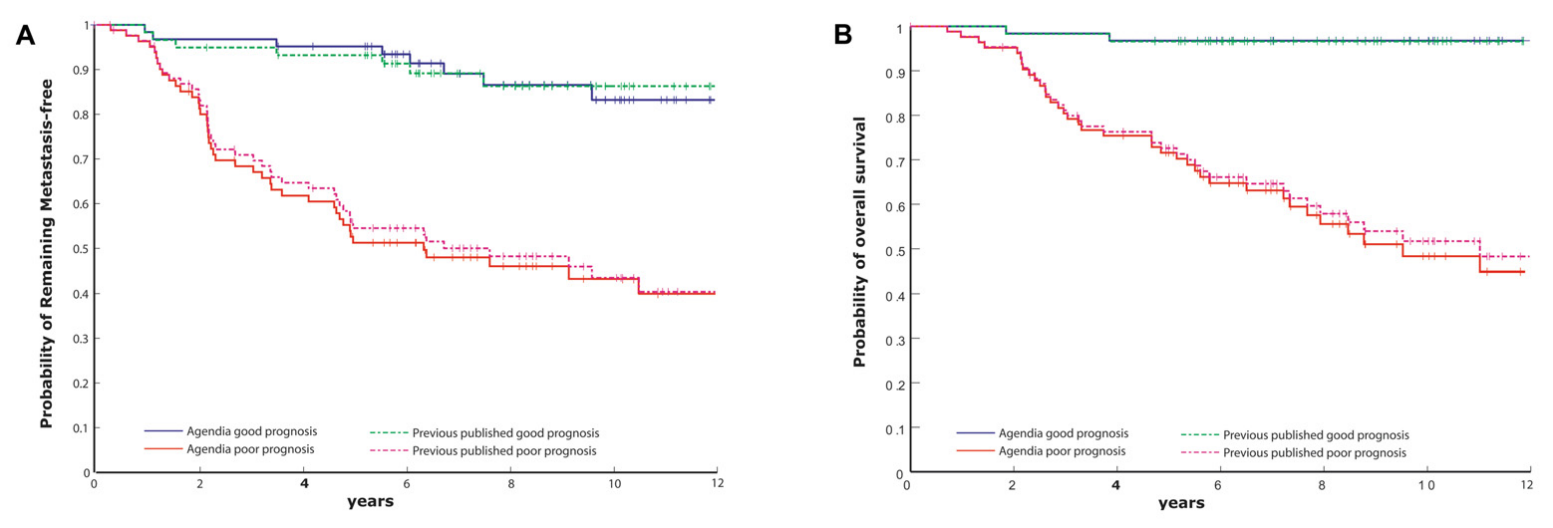
**A**. Kaplan-Meier Analysis of the probability that patients would remain free of distant metastases among 145 patients with lymph-node-negative breast cancer. Blue: Current Good prognosis profile group; green dashed: Good prognosis profile group; Red: Current Poor prognosis profile group; Magenta dashed: previous published data [13] Poor prognosis profile group. **B**. Kaplan-Meier Analysis of the probability of overall survival among 145 patients with lymph-node-negative breast cancer.

As was seen before, Kaplan-Meier curves showed a significant difference in the probability that patients would remain free of distant metastases when classified in the good or poor prognosis profile group (Figure 6A, LogRank p < 0.001). The difference in prediction of probability of overall survival (Figure 6B) between the groups with good or poor prognosis profiles was also highly significant (p < 0.001). The estimated hazard ratio for distant metastases as a first event in the group with a poor prognosis signature versus the group with a good-prognosis signature, over the entire follow up period, was 5.6 (95% CI 2.4 to 7.3, P < 0.0001). This confirms the published data [13] (HR = 5.5, 95%CI 2.5 to12.2, P < 0.001).

When the probability of a patient remaining free of distant metastases was compared between the current result (Figure 6 blue line) and the original analysis (Figure 6 dashed green line) in the good prognosis profile groups, no significant difference was found (logRank p = 0.890). Similarly for those patients in the poor prognosis profile groups (logRank p = 0.794) (Figure 6 red and dashed magenta lines). Equally, there is no significant difference in overall survival of patients grouped by either the current result or the original published result for either prognosis profile group (two results of good prognosis profile group: logRank p = 0.747, two results of poor prognosis profile group logRank p = 0.760, respectively). All results taken together indicate that there is not only a strong correlation between good prognosis and the absence of distant metastasis or death [13], but the findings generated using the more complex microarray platform were nearly perfectly reproduced using the customized mini-arrays, and demonstrate the robustness of the MammaPrint 8-pack mini-array test.

## Discussion

Recently, a 70-gene expression profile was established as a powerful predictor of disease outcome in young breast cancer patients. This profile was generated using microarrays containing 60-mer oligonucleotide probes corresponding to 25,000 transcripts. To enable the use of this prognostic classifier in a high throughput diagnostic setting, a custom-made 8-pack mini-array was developed, which allowed for eight individual simultaneous hybridizations on a single microarray slide (Figure 1). In the present study we tested these custom-made microarrays for use in a diagnostic setting. Sample RNAs from our original studies from which the 70-gene profile was deduced and clinically validated were retrieved, labeled and hybridized to the mini-arrays. Outcome prediction was found to be highly similar to the original data (Figure 3), as well as the probabilities of remaining metastases free and overall survival (Figure 6). There was, however, a small number for which a discrepancy was observed between the current result and that obtained in the original analysis. These were cases for which the 70-gene correlation to the good prognosis template was observed to be very close to the classification threshold (0.4), indicating that minor differences can cause the result for a sample to change from high to low risk classification, or vice versa.

To investigate if these small differences in correlation coefficient were due to technical variation of the customized arrays, 39 samples were labeled, hybridized and analyzed a second time. A high Pearson correlation (0.995) between duplicate samples was observed, indicating very low measurement variability (Figure 4). The diagnostic test was found to be stable over time as well; two samples that were repeatedly labeled and hybridized over a period of 5 months as part of our internal control system showed minimal variation. Most importantly, the outcome of these samples did not gradually shift over time (Figure 5). Differences in outcome between the current and the original data might be due to experimental factors such as different labeling and hybridization protocols, as well as a new reference being used for the microarray hybridizations carried out for this study. Sufficient quantities of this reference stock have now been created for many thousands of hybridizations. Another explanation may be subtle differences in the data processing methods used for the high-throughput diagnostic test, as opposed to those used in the original study. To improve measurement precision in the current analysis, each gene was printed on the mini-array in triplicate, while on the original platform genes were singularly represented. Xdev values were calculated to increase measurement certainty in the initial study, whereas triplicate spotting requires the customized array to use the more robust error-weighted mean log-ratios. The current procedure is therefore considered to be more reliable.

Even though the technical accuracy is extremely high, samples close to the threshold have a higher chance of misclassification than samples further away from the threshold. Based on the known variation in MammaPrint results, a small proportion of MammaPrint samples with indices close to the prediction threshold may have been misclassified. In principle, the chance of a patient with a poor clinical outcome incorrectly being assigned to a good prognosis profile should be minimized. Based on analysis of MammaPrint results generated to date, less than 1.1% of all samples fall into this category. Repeated analysis of borderline samples makes the area of classification uncertainty on either side of the threshold substantially smaller, reducing the proportion of false negative classifications to less than one percent.

The reproducibility of the current test and the similarity of its results to those obtained from the original data demonstrate that it is an excellent tool to predict outcome of disease in breast cancer patients and is highly suitable for a clinical diagnostic clinical setting.

An external validation series by the Transbig consortium using this same customized mini-array, evaluating outcome prediction of 307 patients from 5 European hospitals who were diagnosed with lymph node negative breast cancer before the age of 60 years and who did not receive adjuvant therapy, showed the independent clinical validation of the 70-gene expression profile[21].

## Conclusion

Using the MammaPrint microarray test in the clinical setting will provide more accurate information on recurrence risk as compared to conventional clinical criteria and will thus improve the guidance for the requirement of adjuvant therapy for young women diagnosed with breast cancer. As a direct result, many patients could be potentially spared the side effects and risks of such treatment, improving quality of life and reducing healthcare costs.

## Methods

### Patient samples

One hundred and sixty-two patients with lymph node negative (pN0) breast cancer, age of diagnosis before 55 years, not having received adjuvant therapy and that were part of our previous studies [6,13] were included. All 78 patients that were part of the study in which the 70-gene prognosis profile was established [6], were used to re-establish the 70-gene expression profile. Of the 151 patients used in the clinical validation [13], 145 patients were used, including 61 from the first study. Patients that remained free of disease after initial diagnosis for a period of at least 5 years were assigned to the good prognosis group, i.e. 'good outcome group'; patients that had developed metastasis within 5 years were assigned to the poor prognosis group.

### RNA Isolation and cRNA labelling and hybridization

Aliquots of total RNA of 149 frozen tumor samples was available for this study, for 13 samples (8 out of 78 and 5 of the 145 tumor series, see above) new RNA was isolated from available frozen tumor tissue as described previously [6,13,22]. Two-hundred nanogram total RNA was amplified using the Low RNA Input Fluorescent Labeling Kit (Agilent Technologies). Cyanine 3-CTP or Cyanine 5-CTP (Perkin Elmer) was directly incorporated into the cRNA during in vitro transcription. A total of 200 ng of Cyanine-labeled RNA was co-hybridized with a standard reference to custom 8-pack mini-microarrays (MammaPrint, Agendia) at 60°C for 17 hrs and subsequently washed according to the Agilent standard hybridization protocol (Agilent Oligo Microarray Kit, Agilent Technologies). The reference sample consisted of pooled and amplified RNA of 105 primary breast tumors selected from patients of the clinical validation series [13] in such a way that it had a similar proportional distribution between good and poor profile patients. Sufficient reference material was generated for over 30,000 hybridizations. For each tumor two hybridizations were performed by using a reversal fluorescent dye.

The customized mini-array contained 1,900 60-mer oligonucleotide probes that comprise the 232 prognosis related genes [6] identical to the probes on the original array, including the genes of the 70-gene prognosis classifier, spotted in triplicate. Each array additionally includes 289 probes for hybridization and printing quality control as well as 915 normalization genes. Eight identical MammaPrint arrays are present on a single 1" × 3" slide, allowing for eight individual hybridizations to be performed simultaneously. After hybridization the slides were washed and subsequently scanned with a dual laser scanner (Agilent Technologies). Microarray raw data are available at the European Bioinformatics Institute (EBI) Arrayexpress database;[23] accession number E-TABM-115.

### Data analysis

Fluorescence intensities on scanned images were quantified, values corrected for background non-specific hybridization, and normalized using Feature Extraction software version 7.5.1 (Agilent Technologies). Data was further analyzed using custom algorithms in Matlab version 7.1 (The Mathworks). To obtain an overall expression value for each of the signature genes on the array, an error-weighted mean value was calculated for the three identical probes belonging to the same gene as log_10_ratios. To establish appropriate relative weights, the Rosetta error model was used, which corrects for the uncertainties in individual probe measurements [19,24,25]. Probes were excluded from further calculations if their background corrected intensities were below zero and/or if spots were flagged as non-uniformity outliers as determined by the image analysis software.

### Outcome prediction

Outcome prediction for the 78 tumor samples used in Figure 2 and 3 was performed as described by Van 't Veer et al [6]. In brief, the 'good prognosis template' was (re-)constructed using the average expression for each of the 70 genes in tumors from the 44 'good outcome' patients as determined on the customized mini-array. Subsequently, the expression of the 70 profile genes for each patient was correlated in a leave-one-out cross validation procedure to the 'good prognosis template'. A patient with a cosine correlation to the good prognosis template higher than 0.4 (the previously determined threshold [6]) was assigned to the good-profile group. Patients with a correlation lower than this threshold were assigned to the poor-profile group.

Outcome prediction for the 145 tumor samples used in Figure 6 was performed as described by Van de Vijver *et al*. (13). For each of the 84 tumors from patients that were not included in the original Nature study [6], a correlation coefficient of the 70-gene expression with the template was calculated as described above. For the 61 patients who were included in the original study [6], correlation coefficients were calculated according to the cross-validated classification method using all 231 genes. This approach was originally employed to minimize to some extent the overestimation of the value of the prognosis profile, i.e., no optimization of the number of reporter genes was carried out, as described in the Nature supplementary information by Van 't Veer et al [6]. The only deviation is that 231 instead of a varying number of prognosis correlated genes (range 238 ± 23) were used in the cross-validation procedure since only these 231 genes are present on the mini array. We did show before, however, that the vast majority of the 231 genes were commonly shared by the 78 classifiers generated in the cross-validation procedure [6]. For this subgroup, a patient with a tumor with correlation of the 70 genes higher than the previously determined threshold of 0.55 was assigned to the good prognosis profile, and a correlation of less than 0.55 was assigned to the poor prognosis profile group [13].

### Statistical analysis

Odds ratios were calculated based on a two by two contingency table. P-values associated with odds ratios were calculated by Fisher's exact test. Survival periods of patients were analyzed from the calendar date of surgery to the time of the first event or the date on which data were censored, according to the method of Kaplan Meier. The curves were compared using the log rank test.

## List of abbreviations

OR: Odds Ratio

CI Confidence Interval

Stdev: standard deviation

HR: Hazard ratio

pN0 lymphnode negative

## Authors' contributions

AMG and LJV were involved in the setup of experiments, data analysis, writing of the manuscript and the general overview; AF, set up of experiments and general overview; LJMJD, carrying out experiments and data analysis; ATW, RCFP, NB, JSTLD carrying out experiments, TJB and MOW data analysis, RB set up of experiments and scientific input, LFAW setup of experiments and data analysis. All authors have read and approved the final manuscript
